# *catena*-Poly[[[aqua­(di-2-pyridyl­amine-κ^2^ *N* ^2^,*N* ^2′^)manganese(II)]-μ-5-ferrocenyl­benzene-1,3-dicarboxyl­ato-κ^3^ *O* ^1^,*O* ^1′^:*O* ^3^] methanol monosolvate monohydrate]

**DOI:** 10.1107/S1600536811022781

**Published:** 2011-06-25

**Authors:** Wei Liu, Gang Zhang

**Affiliations:** a Department of Chemistry and Chemical Engineering, Henan University of Urban Construction, Pingdingshan, Henan 467044, People’s Republic of China

## Abstract

In the title coordination polymer, {[FeMn(C_5_H_5_)(C_13_H_7_O_4_)(C_10_H_9_N_3_)(H_2_O)]·CH_3_OH·H_2_O}_*n*_, the Mn^II^ ion has a distorted octa­hedral coordination geometry and is ligated by two N atoms from two di-2-pyridyl­amine mol­ecules, three O atoms from two 5-ferrocenyl­benzene-1,3-dicarboxyl­ate anions and one O atom from a coordinated water mol­ecule. The Mn—O distances range from 2.151 (2) to 2.5093 (19) Å, while the Mn—N distances are 2.226 (2) and 2.248 (2) Å. Each 5-ferrocenyl­benzene-1,5-dicarboxyl­ate anion links to two Mn^II^ ions, resulting in a chain along the *b* axis. A three-dimensional network of N—H⋯O and O—H⋯O hydrogen bonds helps to stabilize the crystal packing.

## Related literature

For the chemical, stereochemical, and electrochemical properties of ferrocene and its derivatives, see: Togni & Hayashi (1995 ▶). In coordination chemistry, there is much inter­est in the introduction of ferrocenyl groups into a ligand framework with the objective of generating materials possessing desired properties, see: Fang *et al.* (2001 ▶); Hudson (2001 ▶); Li *et al.* (2003 ▶). We have recently employed a V-shaped ferrocene-containing dicarboxyl­ate ligand, 5-ferrocenyl­benzene-1,5-dicarb­oxy­lic acid, in the construction of discrete or one-dimensional coordination compounds, see: Li *et al.* (2008 ▶, 2009 ▶). For a related structure, see: Sengupta *et al.* (2001 ▶).
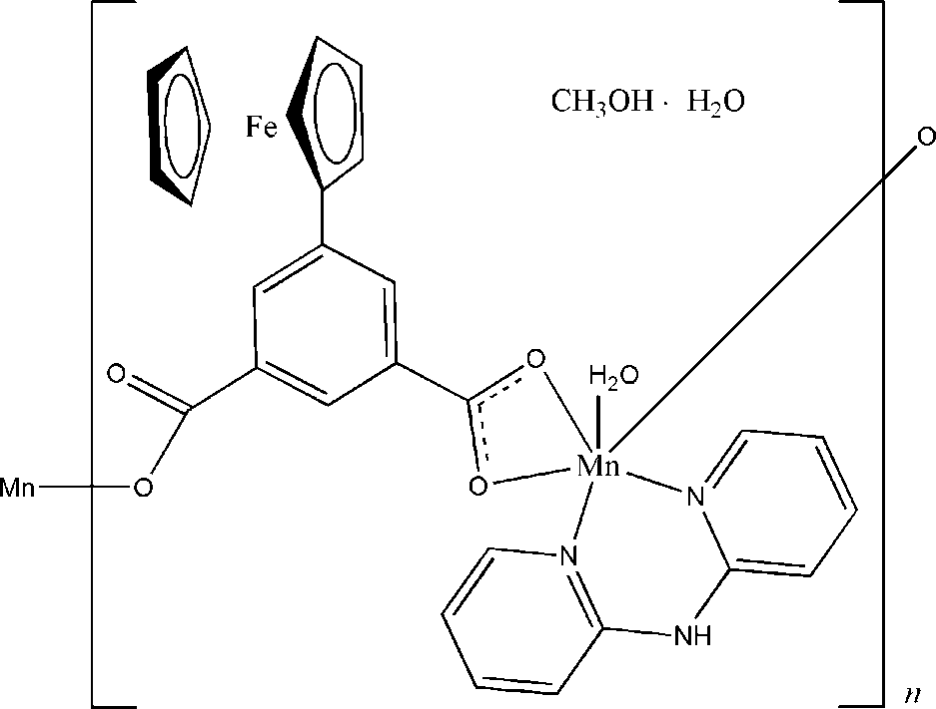


## Experimental

### 

#### Crystal data

[FeMn(C_5_H_5_)(C_13_H_7_O_4_)(C_10_H_9_N_3_)(H_2_O)]·CH_4_O·H_2_O
*M* *_r_* = 642.34
Triclinic, 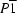

*a* = 9.4550 (19) Å
*b* = 10.174 (2) Å
*c* = 15.153 (3) Å
α = 93.03 (3)°
β = 99.94 (3)°
γ = 94.72 (3)°
*V* = 1427.5 (5) Å^3^
*Z* = 2
Mo *K*α radiation
μ = 1.00 mm^−1^
*T* = 173 K
0.24 × 0.20 × 0.15 mm

#### Data collection

Rigaku Mercury CCD diffractometer
Absorption correction: multi-scan (*CrystalClear*; Rigaku, 2000 ▶) *T*_min_ = 0.798, *T*_max_ = 0.866
8342 measured reflections
5520 independent reflections
4259 reflections with *I* > 2σ(*I*)
*R*_int_ = 0.017

#### Refinement

*R*[*F* ^2^ > 2σ(*F* ^2^)] = 0.038
*wR*(*F* ^2^) = 0.096
*S* = 1.03
5520 reflections
430 parameters
1 restraint
H atoms treated by a mixture of independent and constrained refinement
Δρ_max_ = 0.46 e Å^−3^
Δρ_min_ = −0.46 e Å^−3^

### 

Data collection: *CrystalClear* (Rigaku, 2000 ▶); cell refinement: *CrystalClear*; data reduction: *CrystalClear*; program(s) used to solve structure: *SHELXS97* (Sheldrick, 2008 ▶); program(s) used to refine structure: *SHELXS97* (Sheldrick, 2008 ▶); molecular graphics: *SHELXTL* (Sheldrick, 2008 ▶); software used to prepare material for publication: *SHELXTL*.

## Supplementary Material

Crystal structure: contains datablock(s) I, global. DOI: 10.1107/S1600536811022781/fj2428sup1.cif

Structure factors: contains datablock(s) I. DOI: 10.1107/S1600536811022781/fj2428Isup2.hkl

Additional supplementary materials:  crystallographic information; 3D view; checkCIF report

## Figures and Tables

**Table 1:** Hydrogen-bond geometry (Å, °)

*D*—H⋯*A*	*D*—H	H⋯*A*	*D*⋯*A*	*D*—H⋯*A*
N2—H10⋯O7^i^	0.81 (3)	2.01 (3)	2.816 (4)	173 (3)
O7—H7*A*⋯O3^ii^	0.75 (4)	2.03 (4)	2.693 (3)	148 (4)
O5—H3⋯O1^iii^	0.89 (4)	1.84 (4)	2.731 (3)	177 (4)
O6—H6*A*⋯O4^iii^	0.85 (1)	1.86 (2)	2.685 (3)	162 (4)
O5—H5⋯O6	0.78 (3)	1.88 (3)	2.655 (4)	171 (3)
O7—H7*B*⋯O2	0.86 (6)	1.94 (6)	2.753 (4)	156 (5)

Symmetry codes: (i) 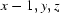; (ii) 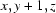; (iii) 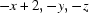.

**Fig. 1.**
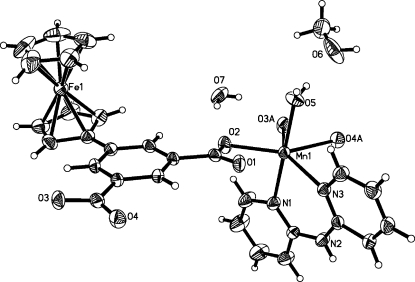
The molecular structure of the title compound, showing the atomic numbering and 30% probability displacement ellipsoids.

## supplementary crystallographic information

### Comment

Ferrocene and its derivates have been extensively studied due to their special chemical, stereochemical, and electrochemical properties (Togni *et al.*, 1995). In the field of coordination chemistry, chemists are strongly interested in introducing ferrocenyl groups into a ligand framework with the objective of generating materials possessing desired properties (Hudson *et al.*, 2001; Fang *et al.*, 2001; Li *et al.*, 2003). Recently, we have been employed a V-shaped ferrocene-containing dicarboxylate ligand 5-ferrocenylbenzene-1,5-dicarboxylic acid to construct discrete or one-dimentional coordination compounds (Li *et al.*, 2008; Li *et al.*, 2009). As our continuing work for this ligand, we report here the synthesis and crystal structure of the title compound.

In the title coordination polymer, {[MnFe(C_18_H_12_O_4_)(C_10_H_9_N_3_)(H_2_O)].CH_3_OH.H_2_O}_n_, the Mn^II^ ion is six coordinated and located in a distorted octahedral geometry ligated by two nitrogen atoms from two 2,2'-dipyridylamine molecules, three oxygen atoms from two 5-ferrocenylbenzene-1,5-dicarboxylate anions and one oxygen atom from one coordinated water molecule (Fig. 1). The O2, O3, O4 and N3 atoms form an equatorial plane (the deviation of the plane being 0.0666 Å), and O5 and N1 occupy the axial position (O5—Mn1—N1 175.65 (9) °). The Mn—O distances range from 2.151 (2) to 2.5093 (19) Å, while Mn—N distances are 2.226 (2) and 2.248 (2) Å, respectively (Table 1). Each 5-ferrocenylbenzene-1,5-dicarboxylate anion links to two Mn^II^ ions resulting a chain along the *b* axis. A three-dimensional network of N—H···O and O—H···O hydrogen bonds (Table 2) help to stabilize the crystal packing.

### Experimental

A methanol solution (5 ml) of 2,2'-dipyridylamine (0.0171 g, 0.1 mmol) was added dropwise to an aqueous solution (5 ml) of MnCl_2_ (0.0198 g, 0.1 mmol), and then a methanol solution (10 ml) of 5-ferrocenylbenzene-1,5-dicarboxylic acid (0.035 g, 0.1 mmol)(Li *et al.*, 2008) was added slowly to the above mixture solution. Finally, the pH value of the mixture was adjusted to about 7 with NaOH aqueous solution, and the resulting orange solution was allowed to slowly evaporate at ambient temperature. Two weeks later, orange block crystals suitable for X-ray single-crystal diffraction analysis were obtained in 48% yield based on Mn. Analysis calculated for C_29_H_29_FeMnN_3_O_7_: C 54.23, H 4.55, N 6.54; found: C 54.46, H 4.50, N 6.42.

### Refinement

Water H atoms and the methanol H atom were located from difference Fourier maps and refined with a *DFIX* restraint of 0.86 (2) Å. Aromatic H atoms were positioned geometrically with C—H = 0.95 Å and constrained to ride on their parent atoms with *U*_iso_(H) = 1.2*U*_eq_(C).

### Crystal data

**Table:**

[FeMn(C_5_H_5_)(C_13_H_7_O_4_)(C_10_H_9_N_3_)(H_2_O)]·CH_4_O·H_2_O	*Z* = 2
*M**_r_* = 642.34	*F*(000) = 662
Triclinic, *P*1	*D*_x_ = 1.494 Mg m^−^^3^
Hall symbol: -P 1	Mo *K*α radiation, λ = 0.71073 Å
*a* = 9.4550 (19) Å	Cell parameters from 2530 reflections
*b* = 10.174 (2) Å	θ = 2.2–29.8°
*c* = 15.153 (3) Å	µ = 1.00 mm^−^^1^
α = 93.03 (3)°	*T* = 173 K
β = 99.94 (3)°	Block, orange
γ = 94.72 (3)°	0.24 × 0.20 × 0.15 mm
*V* = 1427.5 (5) Å^3^	

### Data collection

**Table:**

Rigaku Mercury CCD diffractometer	5520 independent reflections
Radiation source: fine-focus sealed tube	4259 reflections with *I* > 2σ(*I*)
graphite	*R*_int_ = 0.017
ω scans	θ_max_ = 26.0°, θ_min_ = 2.2°
Absorption correction: multi-scan (*CrystalClear*; Rigaku, 2000)	*h* = −11→11
*T*_min_ = 0.798, *T*_max_ = 0.866	*k* = −12→12
8342 measured reflections	*l* = −13→18

### Refinement

**Table:**

Refinement on *F*^2^	Primary atom site location: structure-invariant direct methods
Least-squares matrix: full	Secondary atom site location: difference Fourier map
*R*[*F*^2^ > 2σ(*F*^2^)] = 0.038	Hydrogen site location: inferred from neighbouring sites
*wR*(*F*^2^) = 0.096	H atoms treated by a mixture of independent and constrained refinement
*S* = 1.03	*w* = 1/[σ^2^(*F*_o_^2^) + (0.0422*P*)^2^ + 0.5123*P*] where *P* = (*F*_o_^2^ + 2*F*_c_^2^)/3
5520 reflections	(Δ/σ)_max_ = 0.001
430 parameters	Δρ_max_ = 0.46 e Å^−^^3^
1 restraint	Δρ_min_ = −0.46 e Å^−^^3^

### Special details

**Table:**

Geometry. All e.s.d.'s (except the e.s.d. in the dihedral angle between two l.s. planes) are estimated using the full covariance matrix. The cell e.s.d.'s are taken into account individually in the estimation of e.s.d.'s in distances, angles and torsion angles; correlations between e.s.d.'s in cell parameters are only used when they are defined by crystal symmetry. An approximate (isotropic) treatment of cell e.s.d.'s is used for estimating e.s.d.'s involving l.s. planes.
Refinement. Refinement of *F*^2^ against ALL reflections. The weighted *R*-factor *wR* and goodness of fit *S* are based on *F*^2^, conventional *R*-factors *R* are based on *F*, with *F* set to zero for negative *F*^2^. The threshold expression of *F*^2^ > σ(*F*^2^) is used only for calculating *R*-factors(gt) *etc*. and is not relevant to the choice of reflections for refinement. *R*-factors based on *F*^2^ are statistically about twice as large as those based on *F*, and *R*- factors based on ALL data will be even larger.

### Fractional atomic coordinates and isotropic or equivalent isotropic displacement parameters (Å^2^)

**Table:**

	*x*	*y*	*z*	*U*_iso_*/*U*_eq_	
Fe1	1.65507 (4)	−0.26160 (4)	0.36749 (3)	0.04720 (13)	
Mn1	0.96915 (4)	0.19655 (4)	0.15479 (3)	0.03679 (12)	
O1	0.9617 (2)	−0.05825 (18)	0.11693 (13)	0.0529 (5)	
N1	0.8703 (2)	0.1942 (2)	0.27946 (14)	0.0463 (5)	
C1	1.8357 (6)	−0.3356 (8)	0.3390 (3)	0.110 (2)	
H1	1.899 (5)	−0.372 (5)	0.375 (3)	0.127 (18)*	
O2	1.1190 (2)	0.06247 (17)	0.21790 (14)	0.0554 (5)	
N2	0.6291 (3)	0.2135 (2)	0.21420 (16)	0.0491 (6)	
C2	1.7141 (7)	−0.4008 (5)	0.2832 (3)	0.0996 (15)	
H2	1.663 (4)	−0.485 (4)	0.291 (2)	0.077 (12)*	
O3	1.1239 (2)	−0.63879 (17)	0.22317 (15)	0.0571 (5)	
N3	0.7391 (2)	0.1676 (2)	0.08758 (14)	0.0411 (5)	
C3	1.6431 (5)	−0.3073 (4)	0.2337 (2)	0.0747 (10)	
H3	1.060 (4)	0.160 (4)	−0.011 (3)	0.095 (13)*	
O4	0.9379 (2)	−0.56199 (18)	0.14381 (14)	0.0557 (5)	
C4	1.7244 (5)	−0.1831 (5)	0.2583 (3)	0.0858 (12)	
H4	1.689 (6)	−0.104 (6)	0.235 (4)	0.18 (3)*	
O5	1.0750 (3)	0.2110 (2)	0.04046 (15)	0.0577 (6)	
C5	1.8422 (5)	−0.2019 (8)	0.3250 (3)	0.1049 (17)	
H5	1.106 (3)	0.283 (3)	0.034 (2)	0.051 (9)*	
O6	1.1854 (4)	0.4444 (3)	0.0007 (2)	0.1308 (14)	
C6	1.6383 (4)	−0.2896 (4)	0.4971 (2)	0.0688 (10)	
H6	1.703 (4)	−0.339 (4)	0.541 (3)	0.098 (12)*	
H6A	1.129 (4)	0.476 (4)	−0.041 (2)	0.117*	
O7	1.3625 (3)	0.2353 (4)	0.2701 (2)	0.0782 (8)	
C7	1.6525 (4)	−0.1553 (4)	0.4846 (2)	0.0688 (10)	
H7	1.726 (4)	−0.096 (3)	0.516 (2)	0.073 (10)*	
H7A	1.320 (4)	0.291 (4)	0.255 (3)	0.080 (15)*	
H7B	1.304 (6)	0.168 (6)	0.248 (4)	0.16 (3)*	
C8	1.5133 (4)	−0.3473 (4)	0.4380 (2)	0.0621 (8)	
H8	1.476 (4)	−0.435 (4)	0.436 (3)	0.102 (14)*	
C9	1.5376 (3)	−0.1274 (3)	0.4181 (2)	0.0600 (8)	
H9	1.521 (4)	−0.046 (4)	0.394 (2)	0.092 (13)*	
C10	1.4492 (3)	−0.2464 (3)	0.38753 (18)	0.0456 (6)	
H10	0.551 (3)	0.225 (3)	0.227 (2)	0.061 (10)*	
C11	1.3176 (3)	−0.2627 (2)	0.31824 (17)	0.0395 (6)	
H11	1.553 (4)	−0.320 (4)	0.193 (3)	0.098 (13)*	
C12	1.2505 (3)	−0.3877 (2)	0.28748 (17)	0.0403 (6)	
H12	1.2916	−0.4633	0.3112	0.048*	
C13	1.1256 (3)	−0.4054 (2)	0.22338 (17)	0.0383 (6)	
H13	1.927 (6)	−0.135 (5)	0.358 (3)	0.15 (2)*	
C14	1.0659 (3)	−0.2948 (2)	0.18762 (17)	0.0372 (5)	
H14	0.9805	−0.3055	0.1435	0.045*	
C15	1.2554 (3)	−0.1541 (2)	0.28156 (17)	0.0396 (6)	
H15	1.2987	−0.0677	0.3014	0.048*	
C16	1.1309 (3)	−0.1694 (2)	0.21635 (17)	0.0377 (6)	
C17	1.0656 (3)	−0.0489 (2)	0.18024 (19)	0.0426 (6)	
C18	1.0570 (3)	−0.5430 (2)	0.19399 (19)	0.0435 (6)	
C19	0.9681 (3)	0.1930 (4)	0.3560 (2)	0.0725 (10)	
H19	1.0651	0.1818	0.3508	0.087*	
C20	0.9361 (4)	0.2066 (5)	0.4390 (2)	0.0949 (14)	
H20	1.0086	0.2038	0.4907	0.114*	
C21	0.7960 (4)	0.2246 (5)	0.4475 (2)	0.0861 (12)	
H21	0.7708	0.2354	0.5053	0.103*	
C22	0.6945 (3)	0.2267 (3)	0.3727 (2)	0.0633 (9)	
H22	0.5973	0.2385	0.3772	0.076*	
C23	0.7362 (3)	0.2111 (3)	0.28820 (17)	0.0422 (6)	
C24	0.6225 (3)	0.1900 (2)	0.12293 (18)	0.0412 (6)	
C25	0.4855 (3)	0.1898 (3)	0.06901 (19)	0.0500 (7)	
H25	0.4042	0.2064	0.0955	0.060*	
C26	0.4707 (3)	0.1657 (3)	−0.0214 (2)	0.0588 (8)	
H26	0.3792	0.1665	−0.0588	0.071*	
C27	0.5903 (3)	0.1399 (3)	−0.0581 (2)	0.0594 (8)	
H27	0.5825	0.1213	−0.1210	0.071*	
C28	0.7196 (3)	0.1417 (3)	−0.00215 (19)	0.0522 (7)	
H28	0.8013	0.1237	−0.0279	0.063*	
C29	1.3128 (4)	0.5007 (4)	0.0283 (3)	0.1060 (16)	
H29A	1.3073	0.5944	0.0449	0.159*	
H29B	1.3697	0.4930	−0.0197	0.159*	
H29C	1.3589	0.4579	0.0808	0.159*	

### Atomic displacement parameters (Å^2^)

**Table:**

	*U*^11^	*U*^22^	*U*^33^	*U*^12^	*U*^13^	*U*^23^
Fe1	0.0463 (2)	0.0580 (3)	0.0374 (2)	0.00879 (19)	0.00588 (17)	0.00370 (18)
Mn1	0.0336 (2)	0.0335 (2)	0.0426 (2)	0.00235 (15)	0.00380 (16)	0.00664 (16)
O1	0.0594 (12)	0.0446 (11)	0.0572 (12)	0.0205 (9)	0.0079 (10)	0.0115 (9)
N1	0.0376 (12)	0.0610 (15)	0.0393 (12)	0.0068 (10)	0.0023 (9)	0.0052 (10)
C1	0.080 (3)	0.196 (7)	0.063 (3)	0.077 (4)	0.010 (2)	−0.010 (4)
O2	0.0693 (13)	0.0268 (9)	0.0705 (13)	0.0113 (9)	0.0098 (10)	0.0051 (9)
N2	0.0354 (13)	0.0649 (16)	0.0469 (14)	0.0095 (11)	0.0056 (11)	0.0023 (11)
C2	0.134 (4)	0.095 (4)	0.078 (3)	0.047 (3)	0.030 (3)	−0.012 (3)
O3	0.0541 (12)	0.0265 (9)	0.0891 (15)	0.0068 (8)	0.0058 (11)	0.0087 (9)
N3	0.0377 (11)	0.0407 (12)	0.0438 (13)	0.0029 (9)	0.0043 (9)	0.0035 (9)
C3	0.085 (3)	0.098 (3)	0.0412 (19)	0.020 (2)	0.0085 (18)	−0.0030 (19)
O4	0.0537 (12)	0.0394 (11)	0.0687 (13)	−0.0041 (9)	−0.0004 (10)	0.0040 (9)
C4	0.087 (3)	0.120 (4)	0.050 (2)	−0.012 (3)	0.017 (2)	0.014 (2)
O5	0.0727 (15)	0.0466 (13)	0.0551 (14)	−0.0106 (11)	0.0225 (11)	0.0039 (11)
C5	0.063 (3)	0.189 (6)	0.062 (3)	−0.011 (3)	0.026 (2)	−0.008 (3)
O6	0.122 (3)	0.120 (2)	0.118 (3)	−0.069 (2)	−0.051 (2)	0.076 (2)
C6	0.056 (2)	0.109 (3)	0.0413 (18)	0.006 (2)	0.0053 (15)	0.0189 (19)
O7	0.0418 (13)	0.106 (2)	0.0865 (19)	0.0194 (16)	0.0062 (12)	0.0025 (18)
C7	0.0521 (19)	0.093 (3)	0.054 (2)	0.0016 (18)	−0.0001 (15)	−0.0222 (19)
C8	0.0581 (19)	0.073 (2)	0.0550 (19)	0.0021 (17)	0.0048 (15)	0.0300 (17)
C9	0.0588 (19)	0.055 (2)	0.062 (2)	0.0056 (15)	0.0033 (16)	−0.0125 (16)
C10	0.0444 (15)	0.0498 (16)	0.0431 (15)	0.0056 (12)	0.0088 (12)	0.0021 (12)
C11	0.0415 (14)	0.0389 (14)	0.0395 (14)	0.0052 (11)	0.0098 (11)	0.0028 (11)
C12	0.0441 (14)	0.0287 (13)	0.0494 (15)	0.0083 (11)	0.0080 (12)	0.0085 (11)
C13	0.0406 (14)	0.0295 (13)	0.0468 (15)	0.0059 (10)	0.0114 (11)	0.0045 (11)
C14	0.0387 (13)	0.0306 (13)	0.0438 (14)	0.0077 (10)	0.0090 (11)	0.0054 (10)
C15	0.0478 (15)	0.0274 (12)	0.0453 (15)	0.0008 (11)	0.0143 (12)	0.0034 (11)
C16	0.0451 (14)	0.0276 (12)	0.0442 (14)	0.0082 (10)	0.0153 (11)	0.0063 (10)
C17	0.0519 (16)	0.0312 (14)	0.0515 (16)	0.0099 (11)	0.0227 (13)	0.0096 (12)
C18	0.0479 (16)	0.0297 (13)	0.0544 (17)	0.0018 (11)	0.0142 (13)	0.0037 (12)
C19	0.0440 (17)	0.128 (3)	0.0448 (18)	0.0180 (18)	0.0018 (14)	0.0019 (18)
C20	0.058 (2)	0.183 (5)	0.0413 (19)	0.024 (2)	−0.0028 (16)	0.001 (2)
C21	0.069 (2)	0.148 (4)	0.0433 (19)	0.016 (2)	0.0141 (17)	0.000 (2)
C22	0.0462 (16)	0.096 (3)	0.0490 (18)	0.0079 (16)	0.0111 (14)	0.0012 (17)
C23	0.0372 (14)	0.0453 (15)	0.0433 (15)	0.0031 (11)	0.0054 (11)	0.0041 (12)
C24	0.0402 (14)	0.0355 (14)	0.0461 (15)	0.0031 (11)	0.0019 (12)	0.0069 (11)
C25	0.0394 (14)	0.0560 (17)	0.0529 (17)	0.0061 (12)	0.0007 (12)	0.0116 (13)
C26	0.0496 (17)	0.065 (2)	0.0562 (19)	0.0036 (14)	−0.0079 (14)	0.0146 (15)
C27	0.0612 (19)	0.070 (2)	0.0425 (17)	−0.0005 (15)	−0.0009 (14)	0.0063 (14)
C28	0.0462 (16)	0.0612 (18)	0.0484 (17)	0.0049 (13)	0.0067 (13)	0.0007 (14)
C29	0.064 (2)	0.086 (3)	0.167 (5)	0.002 (2)	0.010 (3)	0.052 (3)

### Geometric parameters (Å, °)

**Table:**

Fe1—C8	2.025 (3)	C6—C7	1.388 (5)
Fe1—C1	2.025 (4)	C6—C8	1.418 (5)
Fe1—C6	2.030 (3)	C6—H6	1.00 (4)
Fe1—C9	2.030 (3)	O7—H7A	0.75 (4)
Fe1—C2	2.033 (4)	O7—H7B	0.86 (6)
Fe1—C7	2.034 (3)	C7—C9	1.408 (5)
Fe1—C10	2.038 (3)	C7—H7	0.92 (3)
Fe1—C3	2.038 (3)	C8—C10	1.425 (4)
Fe1—C5	2.043 (4)	C8—H8	0.93 (4)
Fe1—C4	2.057 (4)	C9—C10	1.421 (4)
Mn1—O5	2.151 (2)	C9—H9	0.94 (4)
Mn1—O2	2.184 (2)	C10—C11	1.475 (4)
Mn1—O3^i^	2.216 (2)	C11—C15	1.391 (3)
Mn1—N3	2.226 (2)	C11—C12	1.393 (3)
Mn1—N1	2.248 (2)	C12—C13	1.386 (3)
Mn1—O4^i^	2.5093 (19)	C12—H12	0.9500
O1—C17	1.243 (3)	C13—C14	1.393 (3)
N1—C23	1.322 (3)	C13—C18	1.505 (3)
N1—C19	1.353 (4)	C14—C16	1.384 (3)
C1—C5	1.386 (8)	C14—H14	0.9500
C1—C2	1.398 (7)	C15—C16	1.392 (4)
C1—H1	0.86 (5)	C15—H15	0.9500
O2—C17	1.265 (3)	C16—C17	1.504 (3)
N2—C23	1.378 (3)	C19—C20	1.347 (5)
N2—C24	1.381 (3)	C19—H19	0.9500
N2—H10	0.81 (3)	C20—C21	1.379 (5)
C2—C3	1.388 (6)	C20—H20	0.9500
C2—H2	0.97 (3)	C21—C22	1.355 (4)
O3—C18	1.263 (3)	C21—H21	0.9500
O3—Mn1^ii^	2.216 (2)	C22—C23	1.408 (4)
N3—C24	1.337 (3)	C22—H22	0.9500
N3—C28	1.349 (3)	C24—C25	1.407 (4)
C3—C4	1.420 (6)	C25—C26	1.359 (4)
C3—H11	0.96 (4)	C25—H25	0.9500
O4—C18	1.239 (3)	C26—C27	1.382 (4)
O4—Mn1^ii^	2.5093 (19)	C26—H26	0.9500
C4—C5	1.402 (6)	C27—C28	1.360 (4)
C4—H4	0.96 (6)	C27—H27	0.9500
O5—H3	0.89 (4)	C28—H28	0.9500
O5—H5	0.78 (3)	C29—H29A	0.9800
C5—H13	1.05 (5)	C29—H29B	0.9800
O6—C29	1.281 (4)	C29—H29C	0.9800
O6—H6A	0.850 (10)		
			
C8—Fe1—C1	126.6 (3)	C1—C5—C4	107.2 (5)
C8—Fe1—C6	40.93 (13)	C1—C5—Fe1	69.4 (3)
C1—Fe1—C6	109.8 (2)	C4—C5—Fe1	70.6 (2)
C8—Fe1—C9	68.16 (15)	C1—C5—H13	122 (3)
C1—Fe1—C9	156.3 (2)	C4—C5—H13	131 (3)
C6—Fe1—C9	67.87 (16)	Fe1—C5—H13	128 (3)
C8—Fe1—C2	109.4 (2)	C29—O6—H6A	120 (3)
C1—Fe1—C2	40.3 (2)	C7—C6—C8	108.1 (3)
C6—Fe1—C2	123.8 (2)	C7—C6—Fe1	70.2 (2)
C9—Fe1—C2	160.71 (19)	C8—C6—Fe1	69.33 (18)
C8—Fe1—C7	68.07 (16)	C7—C6—H6	127 (2)
C1—Fe1—C7	122.37 (18)	C8—C6—H6	125 (2)
C6—Fe1—C7	39.93 (15)	Fe1—C6—H6	127 (2)
C9—Fe1—C7	40.53 (13)	H7A—O7—H7B	102 (4)
C2—Fe1—C7	158.02 (18)	C6—C7—C9	108.4 (3)
C8—Fe1—C10	41.07 (12)	C6—C7—Fe1	69.9 (2)
C1—Fe1—C10	162.3 (2)	C9—C7—Fe1	69.60 (18)
C6—Fe1—C10	69.14 (13)	C6—C7—H7	125 (2)
C9—Fe1—C10	40.89 (12)	C9—C7—H7	127 (2)
C2—Fe1—C10	124.6 (2)	Fe1—C7—H7	126 (2)
C7—Fe1—C10	68.90 (12)	C6—C8—C10	108.6 (3)
C8—Fe1—C3	121.96 (17)	C6—C8—Fe1	69.74 (19)
C1—Fe1—C3	67.35 (18)	C10—C8—Fe1	69.97 (17)
C6—Fe1—C3	158.14 (18)	C6—C8—H8	125 (2)
C9—Fe1—C3	123.82 (15)	C10—C8—H8	126 (2)
C2—Fe1—C3	39.88 (17)	Fe1—C8—H8	130 (3)
C7—Fe1—C3	160.37 (18)	C7—C9—C10	109.0 (3)
C10—Fe1—C3	106.68 (15)	C7—C9—Fe1	69.88 (19)
C8—Fe1—C5	162.1 (2)	C10—C9—Fe1	69.85 (16)
C1—Fe1—C5	39.8 (2)	C7—C9—H9	128 (2)
C6—Fe1—C5	124.67 (17)	C10—C9—H9	123 (2)
C9—Fe1—C5	120.6 (2)	Fe1—C9—H9	124 (2)
C2—Fe1—C5	67.7 (3)	C9—C10—C8	105.9 (3)
C7—Fe1—C5	107.52 (19)	C9—C10—C11	127.3 (3)
C10—Fe1—C5	155.4 (2)	C8—C10—C11	126.8 (3)
C3—Fe1—C5	68.09 (18)	C9—C10—Fe1	69.25 (17)
C8—Fe1—C4	156.93 (16)	C8—C10—Fe1	68.95 (17)
C1—Fe1—C4	66.7 (2)	C11—C10—Fe1	126.56 (18)
C6—Fe1—C4	160.19 (17)	C15—C11—C12	117.5 (2)
C9—Fe1—C4	107.04 (19)	C15—C11—C10	121.4 (2)
C2—Fe1—C4	67.2 (2)	C12—C11—C10	121.1 (2)
C7—Fe1—C4	123.89 (19)	C13—C12—C11	122.1 (2)
C10—Fe1—C4	120.44 (16)	C13—C12—H12	118.9
C3—Fe1—C4	40.59 (16)	C11—C12—H12	118.9
C5—Fe1—C4	39.98 (19)	C12—C13—C14	119.1 (2)
O5—Mn1—O2	92.67 (9)	C12—C13—C18	119.5 (2)
O5—Mn1—O3^i^	88.03 (9)	C14—C13—C18	121.3 (2)
O2—Mn1—O3^i^	87.14 (7)	C16—C14—C13	120.1 (2)
O5—Mn1—N3	100.83 (9)	C16—C14—H14	120.0
O2—Mn1—N3	132.97 (8)	C13—C14—H14	120.0
O3^i^—Mn1—N3	137.62 (7)	C11—C15—C16	121.4 (2)
O5—Mn1—N1	175.65 (9)	C11—C15—H15	119.3
O2—Mn1—N1	87.16 (8)	C16—C15—H15	119.3
O3^i^—Mn1—N1	87.62 (8)	C14—C16—C15	119.8 (2)
N3—Mn1—N1	82.40 (8)	C14—C16—C17	120.7 (2)
O5—Mn1—O4^i^	86.57 (9)	C15—C16—C17	119.5 (2)
O2—Mn1—O4^i^	141.61 (7)	O1—C17—O2	121.2 (2)
O3^i^—Mn1—O4^i^	54.48 (7)	O1—C17—C16	121.4 (2)
N3—Mn1—O4^i^	84.49 (8)	O2—C17—C16	117.4 (2)
N1—Mn1—O4^i^	90.86 (8)	O4—C18—O3	121.0 (2)
C23—N1—C19	117.0 (2)	O4—C18—C13	121.2 (2)
C23—N1—Mn1	128.75 (18)	O3—C18—C13	117.8 (2)
C19—N1—Mn1	113.56 (18)	C20—C19—N1	124.0 (3)
C5—C1—C2	109.4 (5)	C20—C19—H19	118.0
C5—C1—Fe1	70.8 (3)	N1—C19—H19	118.0
C2—C1—Fe1	70.1 (2)	C19—C20—C21	118.6 (3)
C5—C1—H1	125 (3)	C19—C20—H20	120.7
C2—C1—H1	126 (3)	C21—C20—H20	120.7
Fe1—C1—H1	127 (3)	C22—C21—C20	119.5 (3)
C17—O2—Mn1	102.52 (17)	C22—C21—H21	120.3
C23—N2—C24	134.0 (2)	C20—C21—H21	120.3
C23—N2—H10	113 (2)	C21—C22—C23	118.6 (3)
C24—N2—H10	113 (2)	C21—C22—H22	120.7
C3—C2—C1	107.9 (5)	C23—C22—H22	120.7
C3—C2—Fe1	70.3 (2)	N1—C23—N2	121.2 (2)
C1—C2—Fe1	69.5 (2)	N1—C23—C22	122.3 (2)
C3—C2—H2	120 (2)	N2—C23—C22	116.4 (2)
C1—C2—H2	128 (2)	N3—C24—N2	122.1 (2)
Fe1—C2—H2	108 (2)	N3—C24—C25	121.7 (2)
C18—O3—Mn1^ii^	98.82 (16)	N2—C24—C25	116.2 (2)
C24—N3—C28	117.2 (2)	C26—C25—C24	119.4 (3)
C24—N3—Mn1	127.89 (17)	C26—C25—H25	120.3
C28—N3—Mn1	114.26 (18)	C24—C25—H25	120.3
C2—C3—C4	107.4 (4)	C25—C26—C27	119.2 (3)
C2—C3—Fe1	69.9 (2)	C25—C26—H26	120.4
C4—C3—Fe1	70.4 (2)	C27—C26—H26	120.4
C2—C3—H11	128 (2)	C28—C27—C26	118.4 (3)
C4—C3—H11	124 (2)	C28—C27—H27	120.8
Fe1—C3—H11	122 (2)	C26—C27—H27	120.8
C18—O4—Mn1^ii^	85.66 (15)	N3—C28—C27	124.1 (3)
C5—C4—C3	108.1 (5)	N3—C28—H28	117.9
C5—C4—Fe1	69.4 (2)	C27—C28—H28	117.9
C3—C4—Fe1	69.0 (2)	O6—C29—H29A	109.5
C5—C4—H4	131 (4)	O6—C29—H29B	109.5
C3—C4—H4	121 (4)	H29A—C29—H29B	109.5
Fe1—C4—H4	121 (4)	O6—C29—H29C	109.5
Mn1—O5—H3	129 (2)	H29A—C29—H29C	109.5
Mn1—O5—H5	114 (2)	H29B—C29—H29C	109.5
H3—O5—H5	112 (3)		
			
O2—Mn1—N1—C23	−151.6 (2)	C3—Fe1—C7—C6	162.7 (4)
O3^i^—Mn1—N1—C23	121.1 (2)	C5—Fe1—C7—C6	−123.5 (3)
N3—Mn1—N1—C23	−17.6 (2)	C4—Fe1—C7—C6	−164.4 (2)
O4^i^—Mn1—N1—C23	66.7 (2)	C8—Fe1—C7—C9	−81.6 (2)
O2—Mn1—N1—C19	38.1 (2)	C1—Fe1—C7—C9	158.0 (3)
O3^i^—Mn1—N1—C19	−49.2 (2)	C6—Fe1—C7—C9	−119.6 (3)
N3—Mn1—N1—C19	172.1 (2)	C2—Fe1—C7—C9	−169.3 (5)
O4^i^—Mn1—N1—C19	−103.6 (2)	C10—Fe1—C7—C9	−37.3 (2)
C8—Fe1—C1—C5	−163.7 (3)	C3—Fe1—C7—C9	43.1 (6)
C6—Fe1—C1—C5	−120.8 (3)	C5—Fe1—C7—C9	116.9 (3)
C9—Fe1—C1—C5	−40.9 (6)	C4—Fe1—C7—C9	76.0 (3)
C2—Fe1—C1—C5	120.0 (5)	C7—C6—C8—C10	0.3 (4)
C7—Fe1—C1—C5	−78.3 (4)	Fe1—C6—C8—C10	−59.4 (2)
C10—Fe1—C1—C5	155.9 (5)	C7—C6—C8—Fe1	59.7 (2)
C3—Fe1—C1—C5	82.5 (3)	C1—Fe1—C8—C6	77.7 (3)
C4—Fe1—C1—C5	38.2 (3)	C9—Fe1—C8—C6	−80.9 (2)
C8—Fe1—C1—C2	76.4 (4)	C2—Fe1—C8—C6	119.5 (3)
C6—Fe1—C1—C2	119.2 (3)	C7—Fe1—C8—C6	−37.1 (2)
C9—Fe1—C1—C2	−160.9 (4)	C10—Fe1—C8—C6	−119.7 (3)
C7—Fe1—C1—C2	161.8 (3)	C3—Fe1—C8—C6	161.9 (2)
C10—Fe1—C1—C2	35.9 (8)	C5—Fe1—C8—C6	41.9 (8)
C3—Fe1—C1—C2	−37.5 (3)	C4—Fe1—C8—C6	−163.2 (4)
C5—Fe1—C1—C2	−120.0 (5)	C1—Fe1—C8—C10	−162.6 (2)
C4—Fe1—C1—C2	−81.7 (4)	C6—Fe1—C8—C10	119.7 (3)
O5—Mn1—O2—C17	−83.68 (17)	C9—Fe1—C8—C10	38.77 (18)
O3^i^—Mn1—O2—C17	−171.57 (17)	C2—Fe1—C8—C10	−120.8 (2)
N3—Mn1—O2—C17	23.8 (2)	C7—Fe1—C8—C10	82.6 (2)
N1—Mn1—O2—C17	100.67 (17)	C3—Fe1—C8—C10	−78.4 (2)
O4^i^—Mn1—O2—C17	−171.54 (15)	C5—Fe1—C8—C10	161.6 (7)
C5—C1—C2—C3	−0.1 (5)	C4—Fe1—C8—C10	−43.5 (5)
Fe1—C1—C2—C3	60.0 (3)	C6—C7—C9—C10	−0.3 (4)
C5—C1—C2—Fe1	−60.1 (3)	Fe1—C7—C9—C10	59.0 (2)
C8—Fe1—C2—C3	116.9 (3)	C6—C7—C9—Fe1	−59.3 (2)
C1—Fe1—C2—C3	−118.8 (5)	C8—Fe1—C9—C7	81.3 (2)
C6—Fe1—C2—C3	160.2 (3)	C1—Fe1—C9—C7	−52.0 (6)
C9—Fe1—C2—C3	37.7 (8)	C6—Fe1—C9—C7	37.0 (2)
C7—Fe1—C2—C3	−163.7 (4)	C2—Fe1—C9—C7	167.9 (6)
C10—Fe1—C2—C3	73.6 (4)	C10—Fe1—C9—C7	120.3 (3)
C5—Fe1—C2—C3	−82.0 (3)	C3—Fe1—C9—C7	−164.0 (3)
C4—Fe1—C2—C3	−38.6 (3)	C5—Fe1—C9—C7	−81.2 (3)
C8—Fe1—C2—C1	−124.2 (4)	C4—Fe1—C9—C7	−122.6 (3)
C6—Fe1—C2—C1	−80.9 (4)	C8—Fe1—C9—C10	−38.94 (18)
C9—Fe1—C2—C1	156.5 (6)	C1—Fe1—C9—C10	−172.3 (5)
C7—Fe1—C2—C1	−44.8 (8)	C6—Fe1—C9—C10	−83.2 (2)
C10—Fe1—C2—C1	−167.5 (4)	C2—Fe1—C9—C10	47.6 (7)
C3—Fe1—C2—C1	118.8 (5)	C7—Fe1—C9—C10	−120.3 (3)
C5—Fe1—C2—C1	36.8 (3)	C3—Fe1—C9—C10	75.8 (3)
C4—Fe1—C2—C1	80.3 (4)	C5—Fe1—C9—C10	158.5 (2)
O5—Mn1—N3—C24	−158.7 (2)	C4—Fe1—C9—C10	117.1 (2)
O2—Mn1—N3—C24	97.2 (2)	C7—C9—C10—C8	0.5 (3)
O3^i^—Mn1—N3—C24	−59.7 (3)	Fe1—C9—C10—C8	59.5 (2)
N1—Mn1—N3—C24	18.3 (2)	C7—C9—C10—C11	−179.9 (3)
O4^i^—Mn1—N3—C24	−73.3 (2)	Fe1—C9—C10—C11	−120.8 (3)
O5—Mn1—N3—C28	11.4 (2)	C7—C9—C10—Fe1	−59.1 (2)
O2—Mn1—N3—C28	−92.6 (2)	C6—C8—C10—C9	−0.4 (3)
O3^i^—Mn1—N3—C28	110.5 (2)	Fe1—C8—C10—C9	−59.7 (2)
N1—Mn1—N3—C28	−171.5 (2)	C6—C8—C10—C11	179.9 (3)
O4^i^—Mn1—N3—C28	96.87 (19)	Fe1—C8—C10—C11	120.6 (3)
C1—C2—C3—C4	1.1 (5)	C6—C8—C10—Fe1	59.3 (2)
Fe1—C2—C3—C4	60.7 (3)	C8—Fe1—C10—C9	117.4 (3)
C1—C2—C3—Fe1	−59.6 (3)	C1—Fe1—C10—C9	169.8 (5)
C8—Fe1—C3—C2	−82.2 (4)	C6—Fe1—C10—C9	79.9 (2)
C1—Fe1—C3—C2	37.9 (4)	C2—Fe1—C10—C9	−162.8 (2)
C6—Fe1—C3—C2	−49.0 (6)	C7—Fe1—C10—C9	37.0 (2)
C9—Fe1—C3—C2	−165.9 (3)	C3—Fe1—C10—C9	−122.8 (2)
C7—Fe1—C3—C2	161.7 (5)	C5—Fe1—C10—C9	−49.1 (4)
C10—Fe1—C3—C2	−124.5 (3)	C4—Fe1—C10—C9	−80.8 (3)
C5—Fe1—C3—C2	81.1 (4)	C1—Fe1—C10—C8	52.4 (6)
C4—Fe1—C3—C2	117.9 (5)	C6—Fe1—C10—C8	−37.5 (2)
C8—Fe1—C3—C4	159.8 (3)	C9—Fe1—C10—C8	−117.4 (3)
C1—Fe1—C3—C4	−80.1 (4)	C2—Fe1—C10—C8	79.9 (3)
C6—Fe1—C3—C4	−167.0 (4)	C7—Fe1—C10—C8	−80.4 (2)
C9—Fe1—C3—C4	76.1 (3)	C3—Fe1—C10—C8	119.8 (2)
C2—Fe1—C3—C4	−117.9 (5)	C5—Fe1—C10—C8	−166.5 (4)
C7—Fe1—C3—C4	43.8 (6)	C4—Fe1—C10—C8	161.8 (2)
C10—Fe1—C3—C4	117.6 (3)	C8—Fe1—C10—C11	−120.9 (3)
C5—Fe1—C3—C4	−36.9 (3)	C1—Fe1—C10—C11	−68.5 (6)
C2—C3—C4—C5	−1.8 (5)	C6—Fe1—C10—C11	−158.4 (3)
Fe1—C3—C4—C5	58.6 (3)	C9—Fe1—C10—C11	121.7 (3)
C2—C3—C4—Fe1	−60.4 (3)	C2—Fe1—C10—C11	−41.1 (3)
C8—Fe1—C4—C5	−168.3 (5)	C7—Fe1—C10—C11	158.7 (3)
C1—Fe1—C4—C5	−38.1 (3)	C3—Fe1—C10—C11	−1.1 (3)
C6—Fe1—C4—C5	45.7 (7)	C5—Fe1—C10—C11	72.6 (5)
C9—Fe1—C4—C5	117.6 (4)	C4—Fe1—C10—C11	40.9 (3)
C2—Fe1—C4—C5	−82.0 (4)	C9—C10—C11—C15	−7.8 (4)
C7—Fe1—C4—C5	76.3 (4)	C8—C10—C11—C15	171.8 (3)
C10—Fe1—C4—C5	160.1 (3)	Fe1—C10—C11—C15	−98.4 (3)
C3—Fe1—C4—C5	−120.0 (5)	C9—C10—C11—C12	172.6 (3)
C8—Fe1—C4—C3	−48.4 (6)	C8—C10—C11—C12	−7.8 (4)
C1—Fe1—C4—C3	81.9 (3)	Fe1—C10—C11—C12	82.0 (3)
C6—Fe1—C4—C3	165.7 (4)	C15—C11—C12—C13	−0.6 (4)
C9—Fe1—C4—C3	−122.5 (3)	C10—C11—C12—C13	179.0 (2)
C2—Fe1—C4—C3	37.9 (3)	C11—C12—C13—C14	0.8 (4)
C7—Fe1—C4—C3	−163.7 (2)	C11—C12—C13—C18	−179.0 (2)
C10—Fe1—C4—C3	−80.0 (3)	C12—C13—C14—C16	−0.2 (4)
C5—Fe1—C4—C3	120.0 (5)	C18—C13—C14—C16	179.6 (2)
C2—C1—C5—C4	−1.0 (5)	C12—C11—C15—C16	−0.2 (4)
Fe1—C1—C5—C4	−60.8 (3)	C10—C11—C15—C16	−179.8 (2)
C2—C1—C5—Fe1	59.7 (3)	C13—C14—C16—C15	−0.5 (4)
C3—C4—C5—C1	1.7 (5)	C13—C14—C16—C17	−178.3 (2)
Fe1—C4—C5—C1	60.0 (3)	C11—C15—C16—C14	0.7 (4)
C3—C4—C5—Fe1	−58.3 (3)	C11—C15—C16—C17	178.6 (2)
C8—Fe1—C5—C1	47.2 (8)	Mn1—O2—C17—O1	6.6 (3)
C6—Fe1—C5—C1	79.3 (4)	Mn1—O2—C17—C16	−172.10 (18)
C9—Fe1—C5—C1	162.2 (3)	C14—C16—C17—O1	−8.1 (4)
C2—Fe1—C5—C1	−37.3 (3)	C15—C16—C17—O1	174.0 (2)
C7—Fe1—C5—C1	119.9 (3)	C14—C16—C17—O2	170.6 (2)
C10—Fe1—C5—C1	−162.7 (3)	C15—C16—C17—O2	−7.2 (4)
C3—Fe1—C5—C1	−80.5 (3)	Mn1^ii^—O4—C18—O3	−1.4 (3)
C4—Fe1—C5—C1	−117.9 (5)	Mn1^ii^—O4—C18—C13	179.6 (2)
C8—Fe1—C5—C4	165.0 (5)	Mn1^ii^—O3—C18—O4	1.6 (3)
C1—Fe1—C5—C4	117.9 (5)	Mn1^ii^—O3—C18—C13	−179.37 (19)
C6—Fe1—C5—C4	−162.8 (3)	C12—C13—C18—O4	172.6 (3)
C9—Fe1—C5—C4	−79.9 (4)	C14—C13—C18—O4	−7.2 (4)
C2—Fe1—C5—C4	80.6 (3)	C12—C13—C18—O3	−6.4 (4)
C7—Fe1—C5—C4	−122.2 (3)	C14—C13—C18—O3	173.7 (2)
C10—Fe1—C5—C4	−44.8 (6)	C23—N1—C19—C20	0.6 (6)
C3—Fe1—C5—C4	37.4 (3)	Mn1—N1—C19—C20	172.1 (4)
C8—Fe1—C6—C7	−119.3 (3)	N1—C19—C20—C21	−0.8 (7)
C1—Fe1—C6—C7	117.1 (3)	C19—C20—C21—C22	0.7 (7)
C9—Fe1—C6—C7	−37.6 (2)	C20—C21—C22—C23	−0.3 (6)
C2—Fe1—C6—C7	159.9 (3)	C19—N1—C23—N2	−179.9 (3)
C10—Fe1—C6—C7	−81.7 (2)	Mn1—N1—C23—N2	10.1 (4)
C3—Fe1—C6—C7	−164.5 (4)	C19—N1—C23—C22	−0.3 (4)
C5—Fe1—C6—C7	75.2 (4)	Mn1—N1—C23—C22	−170.3 (2)
C4—Fe1—C6—C7	41.1 (6)	C24—N2—C23—N1	6.8 (5)
C1—Fe1—C6—C8	−123.6 (3)	C24—N2—C23—C22	−172.8 (3)
C9—Fe1—C6—C8	81.7 (2)	C21—C22—C23—N1	0.1 (5)
C2—Fe1—C6—C8	−80.8 (3)	C21—C22—C23—N2	179.8 (3)
C7—Fe1—C6—C8	119.3 (3)	C28—N3—C24—N2	178.0 (2)
C10—Fe1—C6—C8	37.6 (2)	Mn1—N3—C24—N2	−12.0 (4)
C3—Fe1—C6—C8	−45.2 (5)	C28—N3—C24—C25	−1.4 (4)
C5—Fe1—C6—C8	−165.5 (3)	Mn1—N3—C24—C25	168.53 (19)
C4—Fe1—C6—C8	160.4 (5)	C23—N2—C24—N3	−5.8 (5)
C8—C6—C7—C9	0.0 (4)	C23—N2—C24—C25	173.6 (3)
Fe1—C6—C7—C9	59.2 (2)	N3—C24—C25—C26	0.3 (4)
C8—C6—C7—Fe1	−59.1 (2)	N2—C24—C25—C26	−179.2 (3)
C8—Fe1—C7—C6	38.0 (2)	C24—C25—C26—C27	1.0 (4)
C1—Fe1—C7—C6	−82.4 (4)	C25—C26—C27—C28	−1.0 (5)
C9—Fe1—C7—C6	119.6 (3)	C24—N3—C28—C27	1.4 (4)
C2—Fe1—C7—C6	−49.7 (6)	Mn1—N3—C28—C27	−169.9 (2)
C10—Fe1—C7—C6	82.3 (2)	C26—C27—C28—N3	−0.2 (5)

Symmetry codes: (i) *x*, *y*+1, *z*; (ii) *x*, *y*−1, *z*.

### Hydrogen-bond geometry (Å, °)

**Table:**

*D*—H···*A*	*D*—H	H···*A*	*D*···*A*	*D*—H···*A*
N2—H10···O7^iii^	0.81 (3)	2.01 (3)	2.816 (4)	173 (3)
O7—H7A···O3^i^	0.75 (4)	2.03 (4)	2.693 (3)	148 (4)
O5—H3···O1^iv^	0.89 (4)	1.84 (4)	2.731 (3)	177 (4)
O6—H6A···O4^iv^	0.85 (1)	1.86 (2)	2.685 (3)	162 (4)
O5—H5···O6	0.78 (3)	1.88 (3)	2.655 (4)	171 (3)
O7—H7B···O2	0.86 (6)	1.94 (6)	2.753 (4)	156 (5)

Symmetry codes: (iii) *x*−1, *y*, *z*; (i) *x*, *y*+1, *z*; (iv) −*x*+2, −*y*, −*z*.
